# Supplementary material for: Transcriptionally induced enhancers in the macrophage immune response to Mycobacterium tuberculosis infection
Source: BMC Genomics. 2019 Jan 22;20:71. doi: 10.1186/s12864-019-5450-6 (PMC6341744; doi:10.1186/s12864-019-5450-6)
Supplement: Supplementary file 1 — Figure S1. Many enhancers respond to M.tb infection with increased eRNA expression. (PDF 80 kb) [file 12864_2019_5450_MOESM1_ESM.pdf]

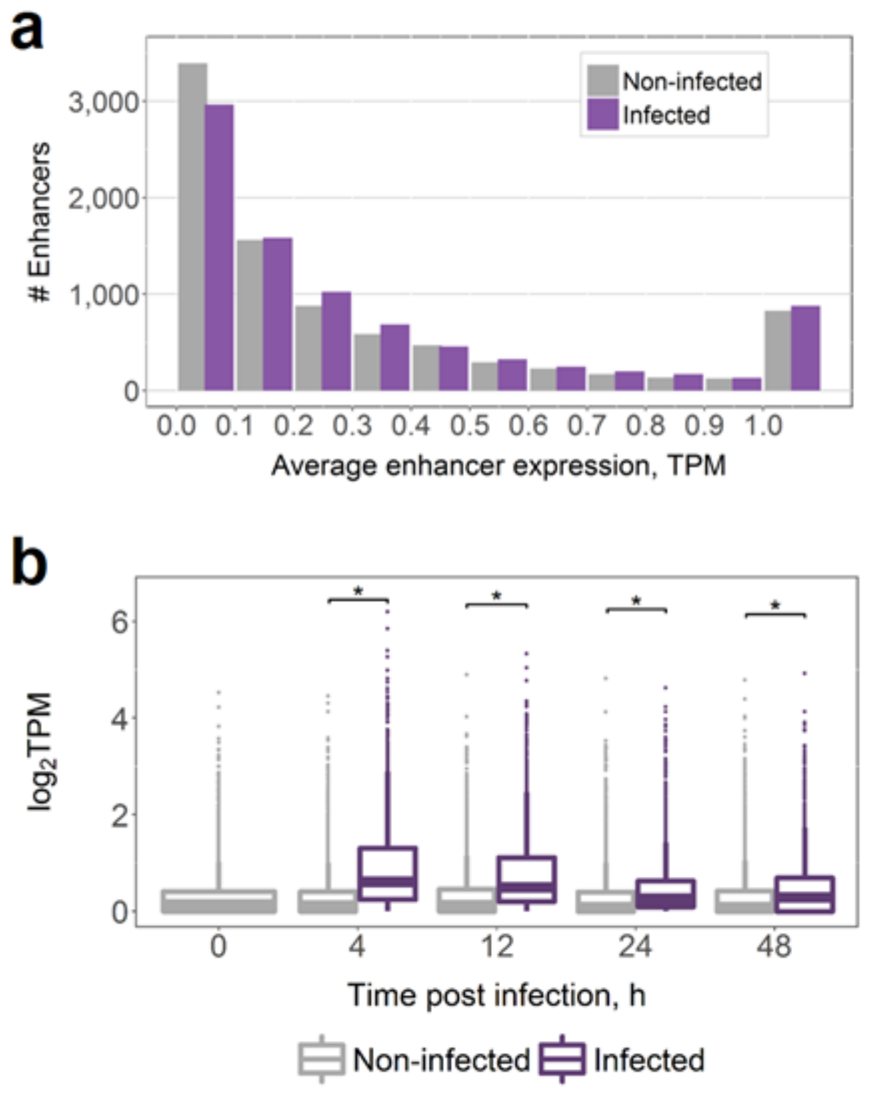

Figure S1. **Many enhancers respond to *M.tb* infection with increased eRNA expression. a** Expression of all 8,667 macrophage enhancer eRNA in non-infected and infected macrophages; each bin includes the left edge. **b** Expression of 2,999 enhancer eRNA associated with up-regulated DEGs; expression in TPM was averaged across replicates, (*) indicate paired two-sided Wilcoxon signed-rank test p-value $< 2.2*10^{-16}$.
