# Supplementary material for: Transcriptionally induced enhancers in the macrophage immune response to Mycobacterium tuberculosis infection
Source: BMC Genomics. 2019 Jan 22;20:71. doi: 10.1186/s12864-019-5450-6 (PMC6341744; doi:10.1186/s12864-019-5450-6)
Supplement: Supplementary file 4 — Figure S2. Higher number of associated enhancers is a concomitant of higher gene expression and immune functions in infected macrophages. (PDF 211 kb) [file 12864_2019_5450_MOESM4_ESM.pdf]

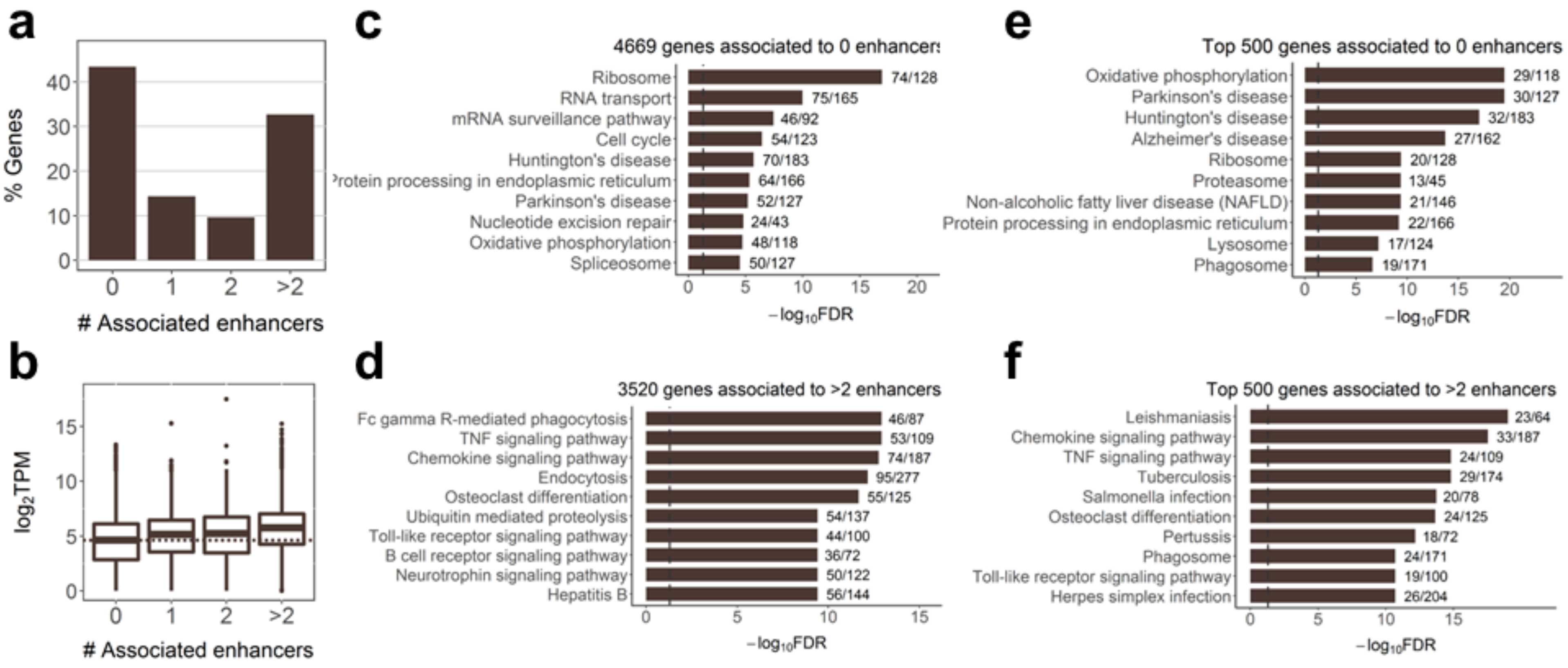

Figure S2. Higher number of associated enhancers is a concomitant of higher gene expression and immune functions in infected macrophages. a, b Percentage and expression of genes associated with different number of enhancers in infected macrophages; expression in TPM was averaged across infected samples, dashed line shows median expression of genes not associated with any enhancer. **c** KEGG pathway maps enriched for genes associated with no transcribed enhancers. **d** KEGG pathway maps enriched for genes associated with more than two transcribed enhancers. **e** KEGG pathway maps enriched for top 500 genes with the highest average expression in infected macrophages among genes associated with no transcribed enhancers. **f** KEGG pathway maps enriched for top 500 genes with the highest average expression in infected macrophages among genes associated with more than two transcribed enhancers. In **c-f,** top 10 maps with the lowest FDR are shown; next to the bars are the numbers of genes in the map covered by our gene list; dashed lines indicate FDR = 0.05.
