# Supplementary material for: Transcriptionally induced enhancers in the macrophage immune response to Mycobacterium tuberculosis infection
Source: BMC Genomics. 2019 Jan 22;20:71. doi: 10.1186/s12864-019-5450-6 (PMC6341744; doi:10.1186/s12864-019-5450-6)
Supplement: Supplementary file 5 — Figure S3. Up-regulated DEGs associated with super enhancers show more infection-specific functions. (PDF 150 kb) [file 12864_2019_5450_MOESM5_ESM.pdf]

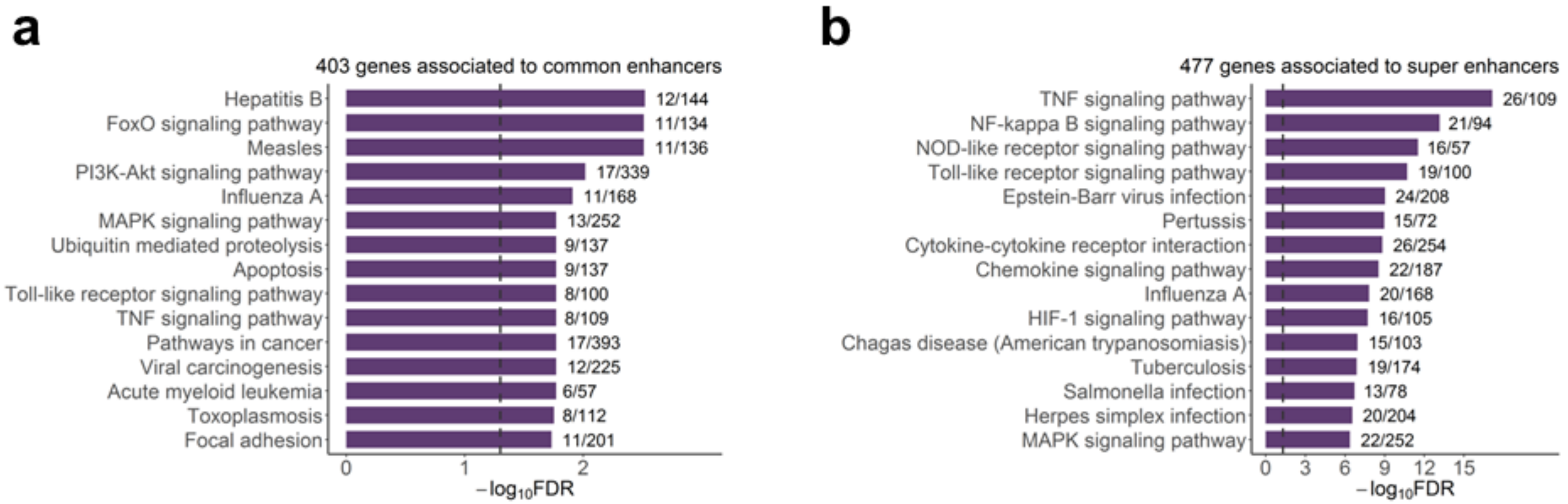

Figure S3. Up-regulated DEGs associated with super enhancers show more infection-specific functions. a KEGG pathway maps enriched for 403 genes associated with transcribed enhancers that do not overlap super enhancer regions. **b** KEGG pathway maps enriched for 477 genes associated with transcribed enhancers overlapping super enhancer regions. In **a** and **b**, top 15 maps with the lowest FDR are shown; next to the bars are the numbers of genes in the map covered by our gene list; dashed lines indicate FDR = 0.05.
