# Supplementary material for: Transcriptionally induced enhancers in the macrophage immune response to Mycobacterium tuberculosis infection
Source: BMC Genomics. 2019 Jan 22;20:71. doi: 10.1186/s12864-019-5450-6 (PMC6341744; doi:10.1186/s12864-019-5450-6)
Supplement: Supplementary file 6 — Figure S4. 257 induced enhancers associated with 263 DEGs up-regulated at 4 h post infection. (PDF 152 kb) [file 12864_2019_5450_MOESM6_ESM.pdf]

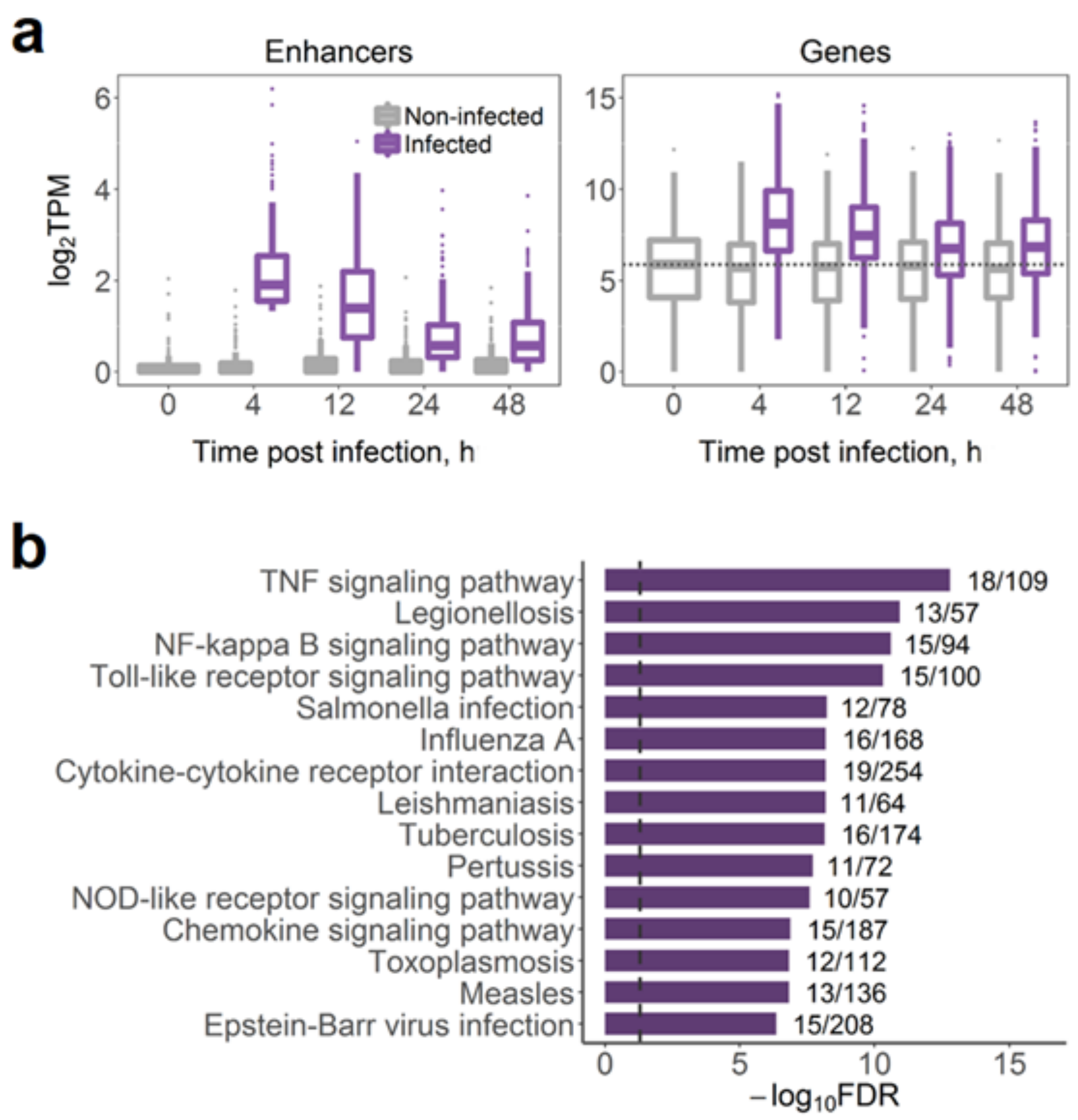

Figure S4. 257 induced enhancers associated with 263 DEGs up-regulated at 4 h post infection. a Expression of enhancer eRNA and genes; dashed line shows median gene expression prior to the infection, expression in TPM was averaged across replicates. **b** Top 15 KEGG pathway maps with the lowest FDR enriched for the genes; next to the bars are the numbers of genes in the KEGG term covered by our gene list; dashed line indicates FDR = 0.05.
