# Supplementary material for: Transcriptionally induced enhancers in the macrophage immune response to Mycobacterium tuberculosis infection
Source: BMC Genomics. 2019 Jan 22;20:71. doi: 10.1186/s12864-019-5450-6 (PMC6341744; doi:10.1186/s12864-019-5450-6)
Supplement: Supplementary file 9 — Figure S5. Expression of the induced enhancers in mouse tissues. (PDF 123 kb) [file 12864_2019_5450_MOESM9_ESM.pdf]

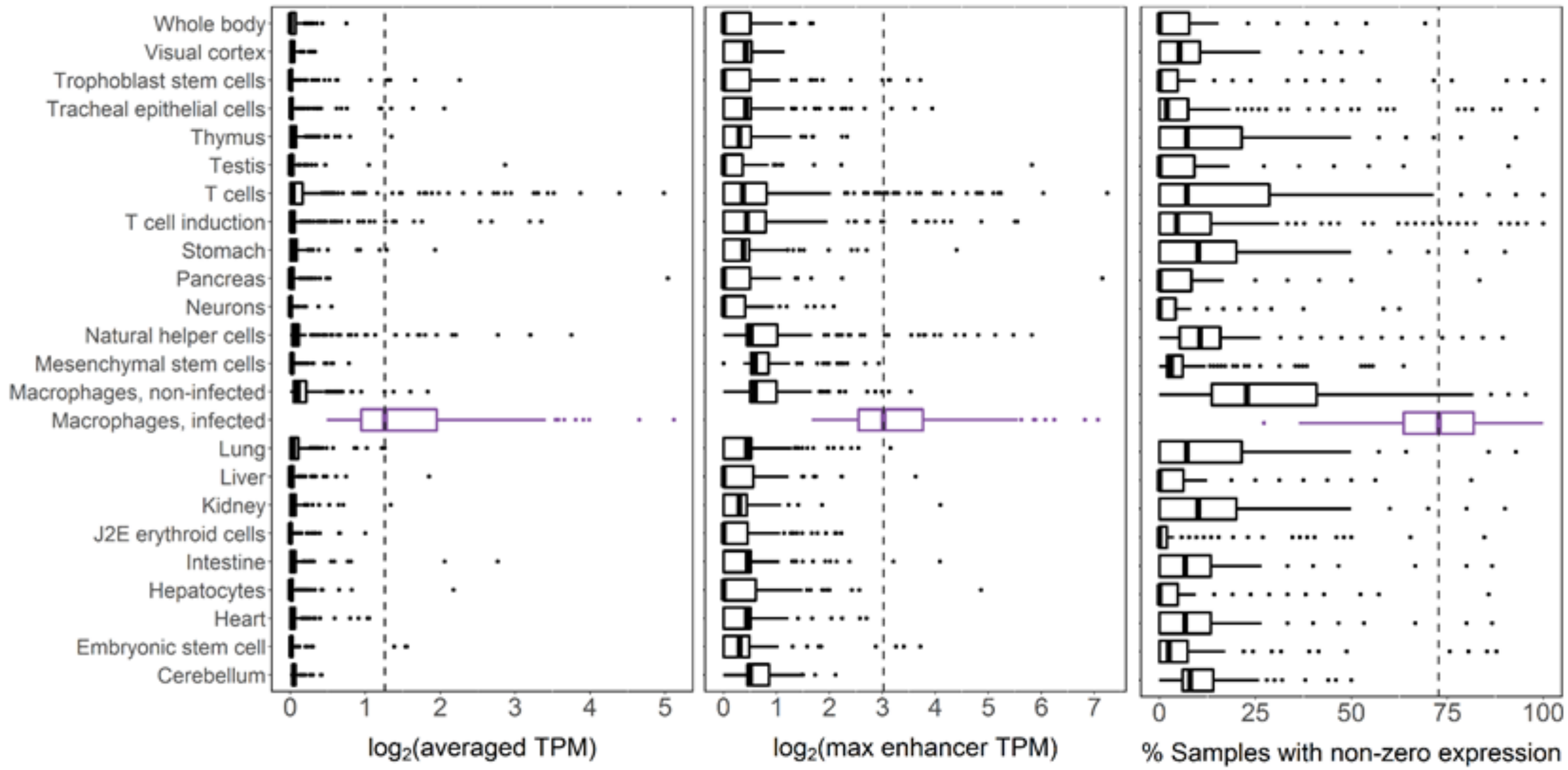

Figure S5. Expression of the induced enhancers in mouse tissues. See Table S4 for the list of non-macrophage samples used. Left panel: expression of each enhancer was averaged across tissue samples. Middle panel: maximum enhancer expression value in each tissue is used. Right panel: percentage of samples with nonzero expression was calculated for each enhancer in each tissue.
