# Supplementary figures and images for: Transcriptionally induced enhancers in the macrophage immune response to Mycobacterium tuberculosis infection
Source: BMC Genomics. 2019 Jan 22;20:71. doi: 10.1186/s12864-019-5450-6 (PMC6341744; doi:10.1186/s12864-019-5450-6)

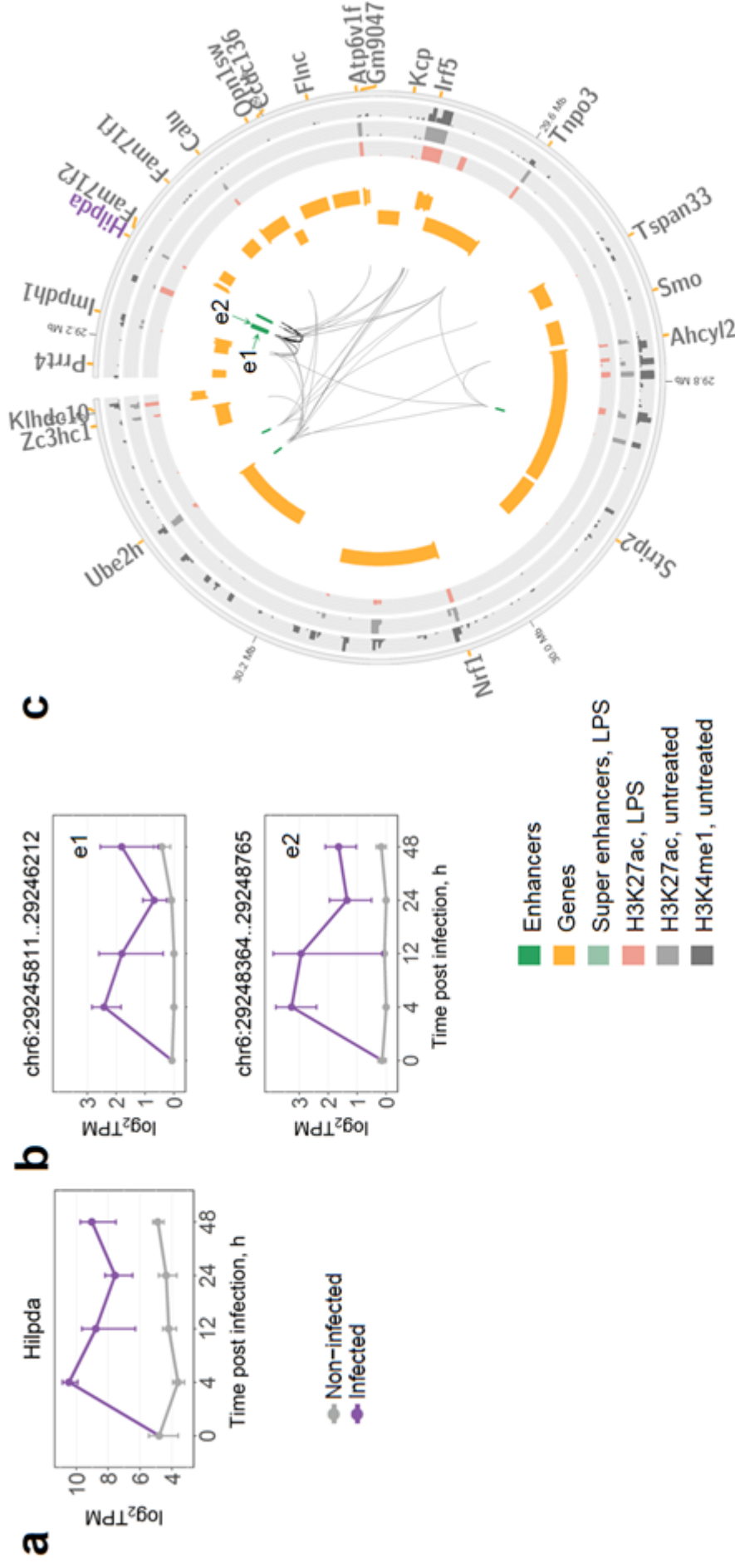

Supplement: Supplementary file 12 — Figure S7. Regulation of Hilpda gene. (PDF 177 kb) [file 12864_2019_5450_MOESM12_ESM.pdf]

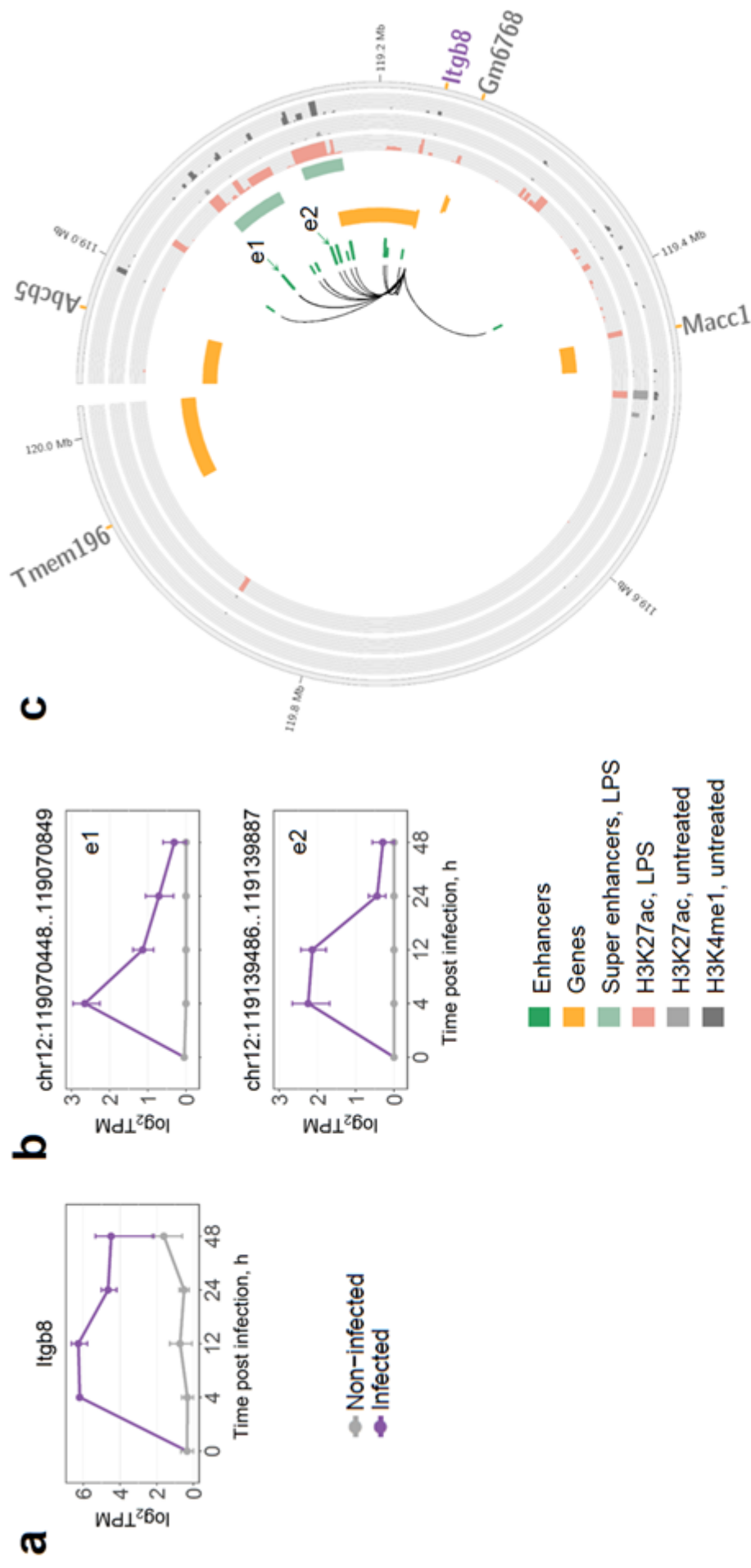

Supplement: Supplementary file 13 — Figure S8. Regulation of Itgb8 gene. (PDF 163 kb) [file 12864_2019_5450_MOESM13_ESM.pdf]

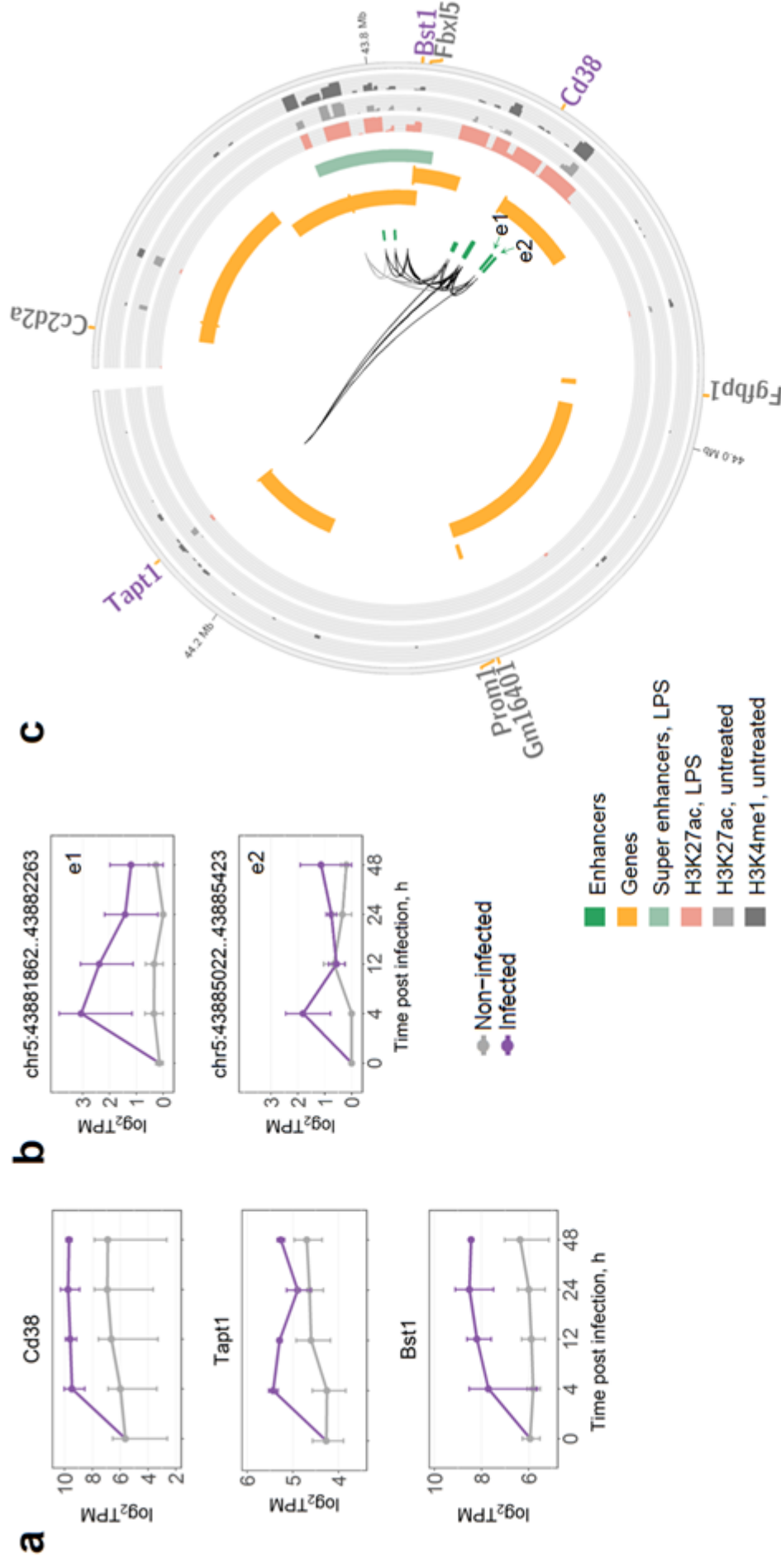

Supplement: Supplementary file 14 — Figure S9. Regulation of Cd38, Bst1, and Tapt1 genes. (PDF 175 kb) [file 12864_2019_5450_MOESM14_ESM.pdf]
