# Supplementary material for: Transcriptionally induced enhancers in the macrophage immune response to Mycobacterium tuberculosis infection
Source: BMC Genomics. 2019 Jan 22;20:71. doi: 10.1186/s12864-019-5450-6 (PMC6341744; doi:10.1186/s12864-019-5450-6)
Supplement: Supplementary file 15 — Figure S10 Regulation of Ccl9, Ccl3, Ccl4, and Wfdc17 genes. (PDF 202 kb) [file 12864_2019_5450_MOESM15_ESM.pdf]

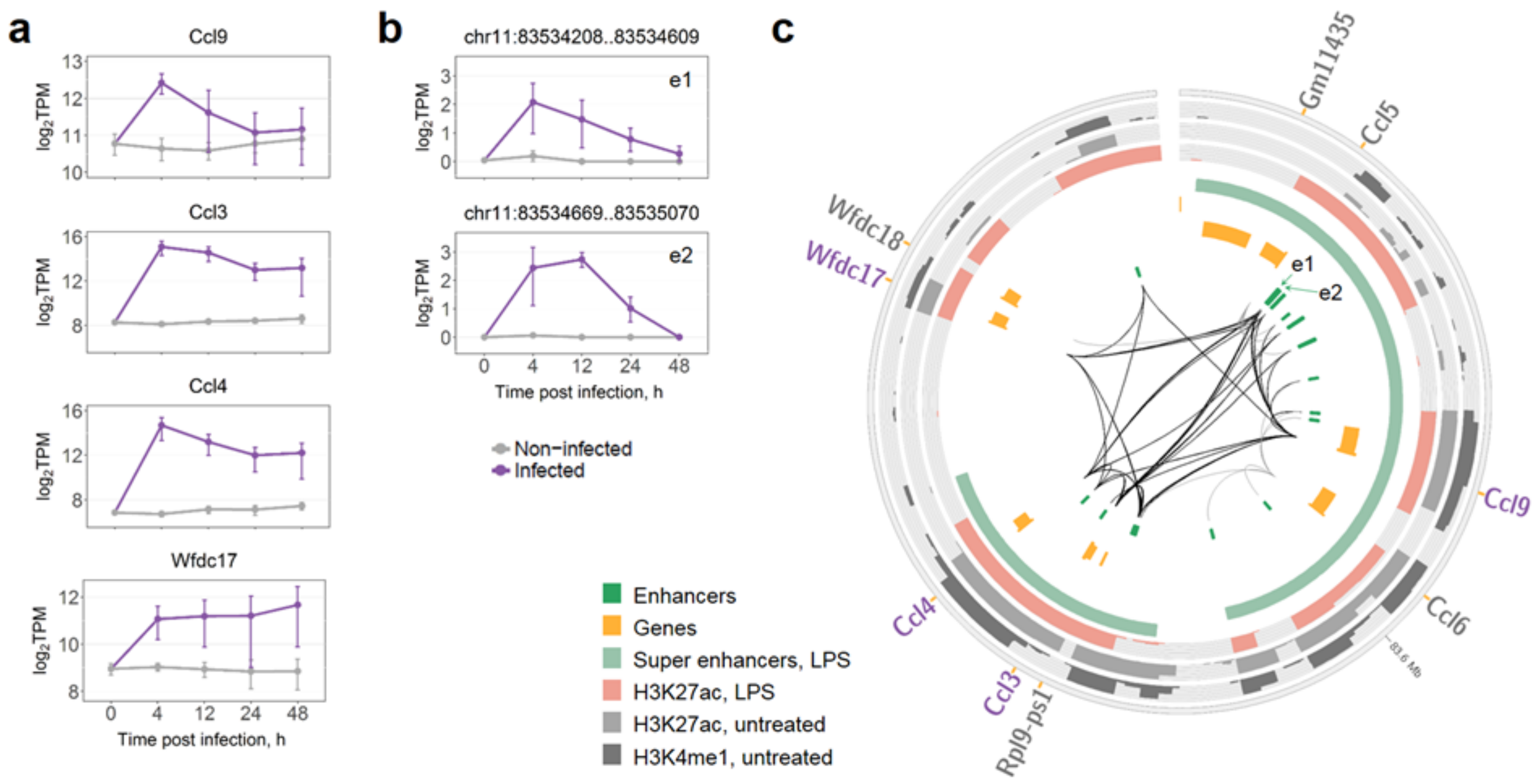

Figure S10. Regulation of *Ccl9*, *Ccl3*, *Ccl4*, and *Wfdc17* genes. a Time course expression of the genes. **b** Time course eRNA expression of associated induced enhancers with the highest average expression at 4 h. In **a** and **b,** data were averaged over replicates and log-transformed, error bars are the SEM. **c** TAD containing the genes and associated enhancers; induced enhancers are shown as longer green blocks. Genes are split into two tracks based on the strand, wide orange marks denote gene promoters. DEGs up-regulated at 4 h are shown in purple and their associations with enhancers are shown as thicker black connections. Super enhancers shown as defined by Hah et al. in LPS-treated macrophages. Histone marks are shown as defined by Ostuni et al. in LPS-treated and untreated macrophages.
