# Supplementary material for: Transcriptionally induced enhancers in the macrophage immune response to Mycobacterium tuberculosis infection
Source: BMC Genomics. 2019 Jan 22;20:71. doi: 10.1186/s12864-019-5450-6 (PMC6341744; doi:10.1186/s12864-019-5450-6)
Supplement: Supplementary file 18 — Figure S12. H3K27ac ChIP-seq peaks. (PDF 49 kb) [file 12864_2019_5450_MOESM18_ESM.pdf]

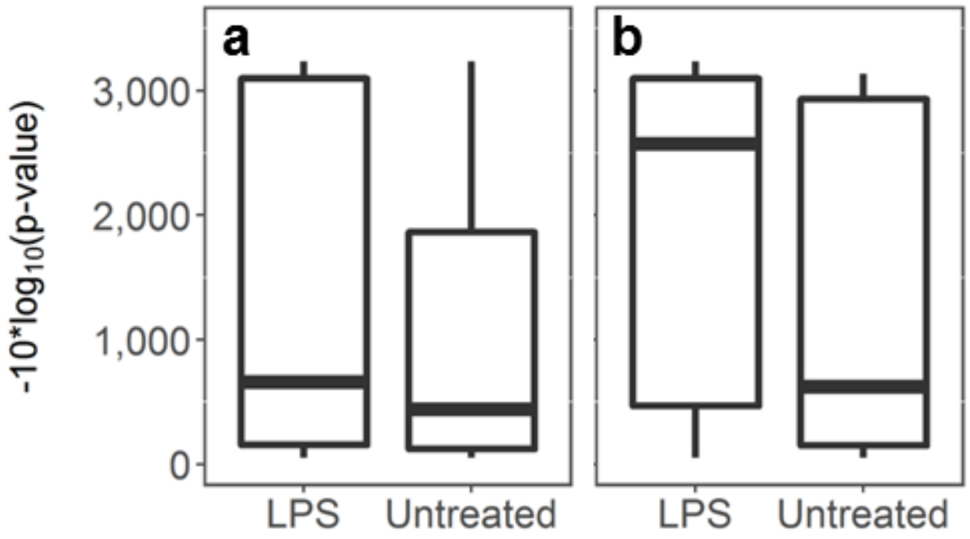

Figure S12. H3K27ac ChIP-seq peaks. Data from Ostuni et al. (Latent Enhancers Activated by Stimulation in Differentiated Cells. Cell. 2013;152:157-171). **a** All significant H3K27ac peaks detected in untreated and LPS-treated samples. **b** A subset of peaks overlapping acquired enhancers.
