# Supplementary material for: Transcriptionally induced enhancers in the macrophage immune response to Mycobacterium tuberculosis infection
Source: BMC Genomics. 2019 Jan 22;20:71. doi: 10.1186/s12864-019-5450-6 (PMC6341744; doi:10.1186/s12864-019-5450-6)
Supplement: Supplementary file 20 — Figure S13. Regulation of Pla2g4a and Ptgs2 genes. (PDF 191 kb) [file 12864_2019_5450_MOESM20_ESM.pdf]

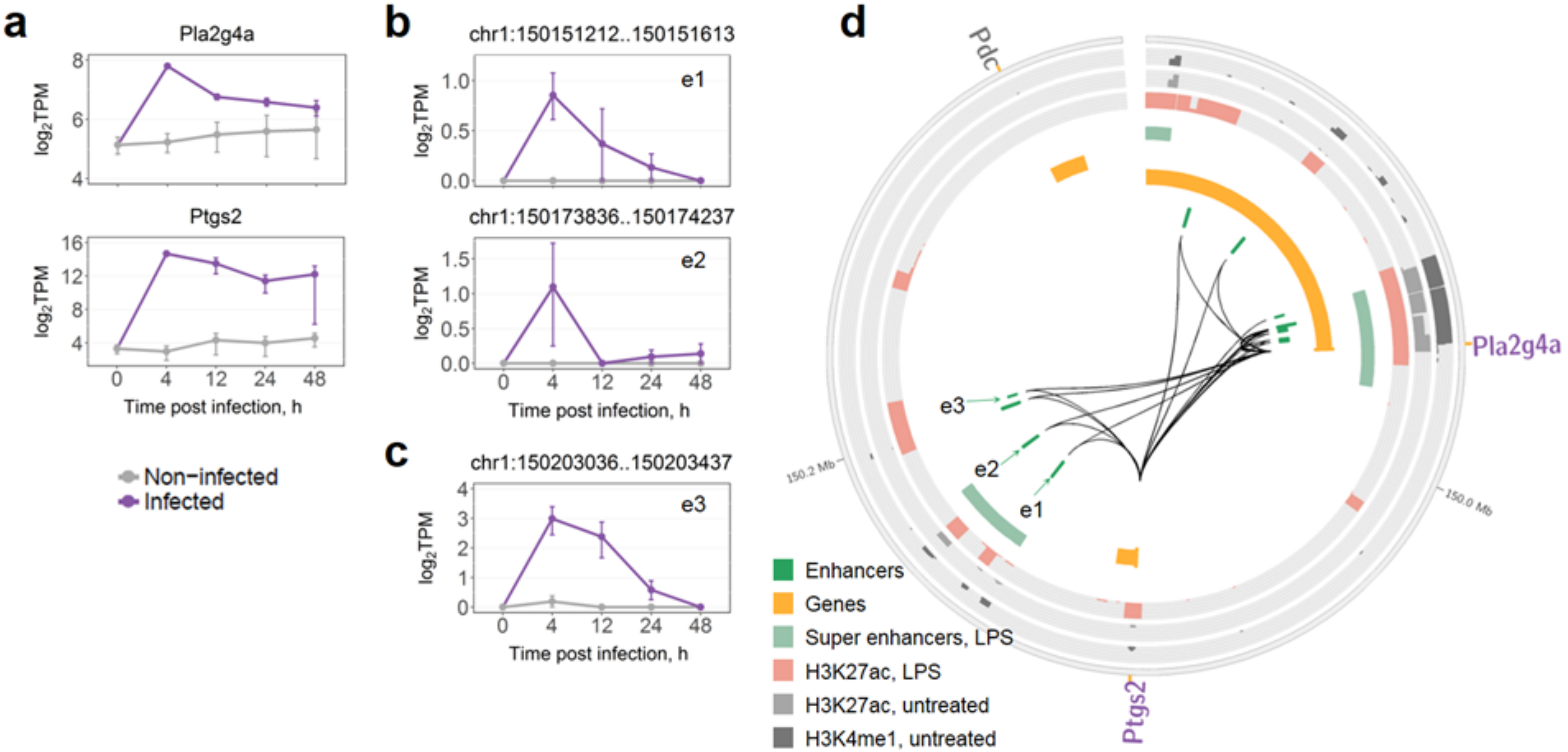

Figure S13. Regulation of *Pla2g4a* and *Ptgs2* genes. a Time course expression of the genes. **b** Time course eRNA expression of associated acquired enhancers with the highest average expression at 4 h. **c** Time course eRNA expression of associated induced enhancer. In **a, b,** and **c,** data were averaged over replicates and log-transformed, error bars are the SEM. **d** TAD containing the genes and associated enhancers; acquired enhancers are shown as longer green blocks. Genes are split into two tracks based on the strand, wide orange marks denote gene promoters. DEGs up-regulated at 4 h are shown in purple. Super enhancers shown as defined by Hah et al. in LPS-treated macrophages. Histone marks are shown as defined by Ostuni et al. in LPS-treated and untreated macrophages.
